# Effect of a mental health education intervention on children’s life satisfaction and self-confidence in rural China

**DOI:** 10.3389/fpsyg.2023.1277139

**Published:** 2023-11-07

**Authors:** Zhengyu Long, Ke Wang, Hui Wang, Weiwei Yao, Chunrong Liu

**Affiliations:** ^1^Institute of Early Education, Beijing Academy of Educational Sciences, Beijing, China; ^2^Faculty of Education, Beijing Normal University, Beijing, China; ^3^Department of Psychology, Faculty of Arts and Sciences, Beijing Normal University at Zhuhai, Zhuhai, China; ^4^Faculty of Psychology, Beijing Normal University, Beijing, China

**Keywords:** mental health education, life satisfaction, self-confidence, rural children, randomized controlled trial

## Abstract

Children living in rural areas may potentially experience low levels of life satisfaction and face challenges in developing self-confidence. The purpose of the current study was to examine the impact of a mental health education intervention on the life satisfaction and self-confidence of children residing in rural areas of China. A total of 1,001 children from grades 4 to 6 were randomly assigned to an intervention group (475 children, 250 boys, *M* = 11.57 years, *SD* = 1.082 years) and a control group (526 children, 279 boys, *M* = 11.38 years, *SD* = 0.980 years). Over 16 weeks, the intervention group received a mental health education program, while the control group did not. The levels of life satisfaction (including five dimensions: family, school, environmental, friends, and self-satisfaction) and self-confidence (including three dimensions: self-efficacy, self-assurance, and self-competence) were rated by all children at baseline and post-intervention. Results from paired samples *t*-test showed that post-intervention, the intervention group exhibited significant improvements in the areas of family, environmental, school, and self-satisfaction as well as self-efficacy, self-assurance, and self-competence. However, there was no significant improvement in friend satisfaction. Conversely, the control group showed decreases in school, environmental, and friend satisfaction, along with decreases in self-efficacy, self-assurance, and self-competence. No significant change was observed in family and self-satisfaction in this group. These findings emphasize the importance of implementing mental health education interventions for rural children, who are at risk for low life satisfaction and self-confidence. Some specific recommendations are provided for policymakers and practitioners.

## 1. Introduction

Over the past several decades, China has experienced dramatic economic changes, accompanied by rapid urbanization. While these changes have promoted the development of many aspects of Chinese society, they have also magnified disparities between urban and rural living standards. The diminished emphasis on rural education, paired with challenges in attracting educators to these regions, has considerably impacted the quality of educational opportunities available to children (Pan and Ye, 2017). Moreover, the urbanization process attracts numerous rural adults to migrate to urban areas, in order to increase family income. As a result, many rural communities are currently characterized by a higher concentration of older residents and children who have been left behind (Mohabir et al., 2017). These left-behind children lack parental monitoring, supervision, and support, which can make them more susceptible to heightened psychosocial distress and poor life satisfaction (Wang et al., 2017; Rao et al., 2019). Although altering a student’s living conditions may be challenging, researchers and educators can invest efforts to enhance children’s mental health, thereby facilitating a more fulfilling life for them. This study aimed to examine the effect of a mental health education program on life satisfaction and self-confidence levels among children residing in rural regions of China, recognized as international poverty-stricken areas.

### 1.1. Rural children’s early development

In rural China, the primary sources of family income continue to be agricultural production. Due to the limited land resources allocated to each family and the unpredictability of natural climatic conditions, rural families’ annual incomes are relatively low. Low SES encompasses more than economic constraints; it can significantly impact children’s developmental trajectories and overall mental health. For example, Yoshikawa et al. (2012) found that children from low-SES backgrounds had higher levels of psychological stress and were more likely to develop emotional problems and mental health disorders. Similarly, Evans and Kim (2013) showed that children living in low-income households experienced higher levels of environmental stressors and were at a higher risk of developing psychological problems. Low SES not only intensifies the psychological distress in children, but also negatively impacts their overall quality of life, leading to decreased life satisfaction.

### 1.2. Rural children’s life satisfaction

Life satisfaction is an important aspect of children’s positive development. Life satisfaction, an aspect of subjective well-being, is a multi-dimensional concept that encompasses a person’s contentment with various facets of life, including school, family, friends, environment, and self (Diener et al., 1985). A child’s life satisfaction is significantly influenced by their family’s SES, with children from low SES families often experiencing a variety of environmental stressors that can negatively impact their perception of life satisfaction (Bradley and Corwyn, 2002; Evans and Kim, 2013). Such children may confront challenges such as housing instability, food insecurity, and limited access to educational resources, which can lower their satisfaction with school and the environment (Evans, 2004; Yoshikawa et al., 2012).

Moreover, rural children typically receive less emotional and educational support from their parents (Wen and Lin, 2012). Rural areas in China refer to places where the population is primarily engaged in agricultural production (National People’s Congress, 2021). Compared to towns and cities, rural areas have a more dispersed population distribution, less advanced infrastructure, and lag behind in economic development (Wiggins and Proctor, 2001). Children living in these under-developed rural areas face numerous challenges, chiefly poverty problems and being left behind, both of which significantly impact physical and mental development (Hu et al., 2014; Feng et al., 2015; Li et al., 2020). Poverty takes a severe toll on Chinese children’s growth and health. Those raised in impoverished conditions exhibit high rates of developmental delays across communication, gross motor, fine motor, problem-solving, and personal-social domains (Wei et al., 2015). Mental health is also impacted, with higher risks of depression, anxiety, and sleep disorders compared to economically disadvantaged children (Fang et al., 2015).

Moreover, many rural children are left behind as parents migrate to cities for work. In 2021, 7.8 million primary school students were left-behind children across China. Frequently raised by grandparents or forced into self-reliance, these children endure neglect of their emotional, physical, social, and educational needs (Wen et al., 2021). This neglect elevates their risk for mental health problems (Hu et al., 2014; Liang et al., 2017). One systematic evaluation, for instance, found that the incidence of serious mental illness among left-behind youth was 2.7 times higher than non-left-behind peers (Wu et al., 2019). More importantly, impoverished children living in rural areas tend to have lower life satisfaction. Rural children may encounter decreased life satisfaction due to fewer educational and recreational opportunities at home (Duncan et al., 2011). Additionally, rural children often suffer from reduced parental involvement, which can further erode their satisfaction with family and self (Hill et al., 2004). In conclusion, poverty has the potential to significantly impact children’s satisfaction in various crucial areas of life. Therefore, interventions that are specifically designed to enhance rural children’s life satisfaction are of utmost importance.

### 1.3. Rural children’s self-confidence

Moreover, self-confidence, another vital component of positive child development, entails an individual’s belief in their own self-worth and their capacity to achieve life goals (Bandura, 1997). This concept includes several dimensions, such as self-efficacy (belief in one’s capacity to attain goals), self-assurance (confidence in one’s worth or abilities), and self-competence (perceived ability to execute tasks; Merenda, 2021). Prolonged exposure to poverty-related stressors often results in diminished self-efficacy and self-esteem, both integral to self-confidence (Evans, 2004). For example, Yoshikawa et al. (2012) found that children living in poverty often reported lower self-confidence due to heightened psychosocial stress and diminished opportunities for achievement. Additionally, low levels of parental involvement in education have been linked to a decrease in children’s confidence regarding academic accomplishment (Maryani et al., 2018). Evidence suggests that limited access to educational resources and extracurricular activities can also hinder children’s development of self-competence and self-efficacy (Evans, 2004).

### 1.4. Interventions targeting children’s life satisfaction and self-confidence

Mental health intervention is a term that refers to any action or activity that aims to improve the mental health and well-being of individuals or groups who are experiencing or at risk of mental health problems (World Health Organization, 2001). It is noteworthy that mental health interventions can serve as potent mitigating forces to enhance life satisfaction and self-confidence among children in economically disadvantaged conditions (Emmers et al., 2021; Li and Hesketh, 2023). Research shows that mental health interventions, when tailored to children’s unique experiences and contexts, can effectively improve their life satisfaction and self-confidence (Weare and Nind, 2011). For example, school-based mental health programs that focus on building resilience, emotional awareness, and social skills have been shown to enhance children’s life satisfaction and self-confidence (Durlak et al., 2011). These programs, which include various strategies such as cognitive-behavioral therapy, mindfulness training, and social–emotional learning, can address the adversities that rural children often face. They offer a buffer against the negative impacts of these adversities on children’s life satisfaction and self-confidence.

However, these interventions are not without their limitations. The heterogeneity of intervention strategies, combined with variations in implementation and assessment methodologies, can make it challenging to derive definitive conclusions about their effectiveness (Weisz et al., 2013). Furthermore, most of the intervention studies have been conducted in high-income contexts, limiting their generalizability to low-SES settings (Lund et al., 2018). This limitation is particularly relevant for children residing in impoverished rural areas in China. Due to remote locations and a dearth of high-quality educational resources, many students in these areas have limited access to mental health-related knowledge. Finally, for rural children living in disadvantaged environments, there is a universal need for mental health promotion. This support is crucial in helping them alleviate feelings of loneliness, anxiety, depression, and low self-esteem, which can stem from risk factors like low SES and parental migration to urban areas for employment (Guan and Deng, 2019). However, it’s noteworthy that most previous intervention studies have typically involved the selection of a limited number of children through random sampling, rather than implementing interventions across entire school populations (Durlak et al., 2011). Indeed, schools play a pivotal role in promoting mental health, given their extensive reach within a significant portion of the population. By offering a comprehensive mental health education curriculum, school-based universal interventions have the potential to improve the overall school environment and enhance students’ quality of life (Fazel et al., 2014). Thus, there is a need for school-based interventions that adapt their content to ensure it is comprehensible for rural students, thereby enhancing the overall intervention efficacy.

### 1.5. Current study

The aim of the current study was to examine the impact of a mental health education program on life satisfaction and self-confidence among children living in rural China. Considering the limited comprehension abilities of younger students, we intend to include all upper-grade students (i.e., those in grades 4–6) from each school as the target of our intervention. By adopting this approach, we aimed to assess the effectiveness of the school-based universal intervention in enhancing the overall mental health of all 4^th^ to 6^th^-grade students in the intervention group. It was hypothesized that children in the intervention group would experience significant improvements in their levels of life satisfaction and self-confidence, whereas no substantial changes would be observed among children in the control group that did not receive any specific intervention.

## 2. Method

### 2.1. Participants

A total of 1,001 children from grades 4 to 6 (*M* = 11.47 years, *SD* = 1.033 years, 529 boys, 472 girls) were recruited from 54 primary schools located in three poverty counties in China: Jingyuan and Jingtai in Gansu Province, and Tailai in Heilongjiang Province. Participants were recruited through contact with educational departments in multiple rural counties. Eventually, these three counties agreed to participate in this study. These counties were previously designated as international poverty-stricken areas, with the average annual income per person falling below 1,500 RMB (approximately 210 USD). However, through concerted poverty alleviation efforts by the Chinese government, these counties were successfully lifted out of poverty by the end of 2020. However, this study took place from September 2019 to June 2020, during which period these counties were still classified as impoverished.

### 2.2. Mental health education program

The mental health education program utilized in this research was developed by the research team, drawing inspiration from the ‘Health & Wellness’ curriculum crafted by McGraw Hill in the USA. The original program provides a comprehensive and easily teachable core curriculum that promotes healthy living among students. We adapted it specifically to resonate with rural children in China.

The intervention program includes teaching plan sets for teachers, PowerPoint lessons, teaching materials, and in-class workbooks for student use. Adopting a child-centered perspective, the project emphasizes progressive in-depth learning. The curriculum content is integrated based on dimensions related to life satisfaction (family, school, environment, self, friends) as well as themes of self-cognition. The course consists of four modules: Module 1 focuses on Emotion and Mental Health; Module 2 delves into Self-awareness and Personal Growth; Module 3 addresses Social Relationships, encompassing Family, Peers, and Community; and Module 4 centers on the Pursuit of a Fulfilling Life. Specific curriculum content is tailored according to the comprehension abilities of students in grades 4–6.

### 2.3. Procedure

**Figure 1:**
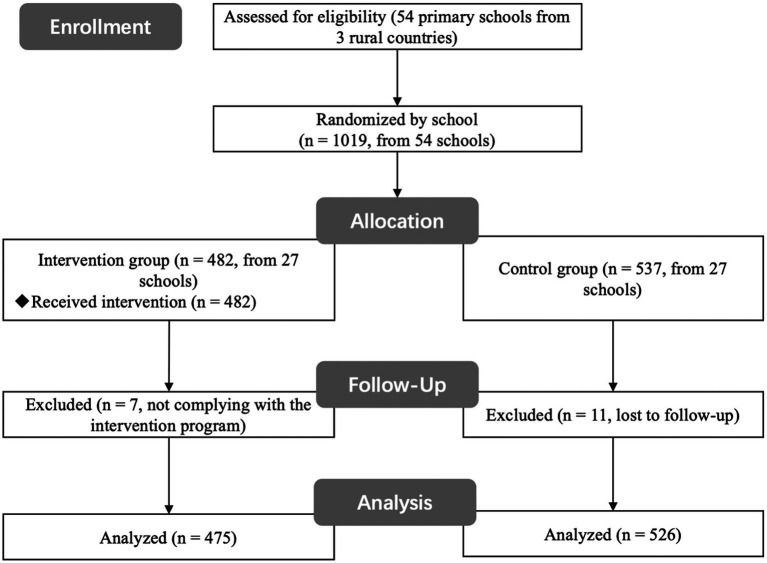
The schematic diagram represents data collection points for the intervention group and the control group.

The study procedure was approved by the university’s institutional review board (IRB). Before participating, children provided signed informed consent. As this intervention adopted a school-based universal intervention approach, all primary schools overseen by the three participating counties were randomly assigned to either the intervention group or the control group. This arrangement ensured that all students from grades 4 to 6 in these schools participated in the project simultaneously. This intervention study was conducted in three stages, detailed below (Figure 1):

Stage 1: Baseline Survey (September 2019). At the beginning of the school year, research assistants conducted a baseline survey with all participating students. Each child completed online questionnaires to report their demographic information (e.g., family income, maternal and paternal educational levels) and to assess their life satisfaction and self-confidence. A total sample of 1,019 students was acquired during this baseline survey.

Stage 2: Implementation of the Mental Health Education Intervention (September 2019–June 2020). The whole project lasted 2 years (September 2019–June 2021). In the first year, all primary schools in the intervention group underwent the mental health intervention program, while those in the control group did not receive any intervention and were treated as waiting groups. All the waiting groups were scheduled to receive the intervention in the second year. According to the study aims, we focused solely on the data collected from the first year.

Regarding the scheduling of the intervention sessions, due to the majority of schools being unable to arrange a weekly intervention session based on their existing teaching schedules, it was decided to conduct the sessions bi-weekly. Given that a typical school term in China spans 16 weeks, a total of 16 sessions were conducted (the first term being from September to December 2019, and the second from March to June 2020). Each session, lasting 45 min, covered topics including self-recognition, emotion regulation and mental health, family and parent–child relationships, peer relationships, friendships, and social life. The final session provided an overview without a designated curriculum.

All intervention sessions took the form of classroom-based interventions, resulting in high fidelity from the students. All sessions were administered by regular classroom teachers. Prior to initiating the intervention, teachers underwent intensive training for a week. The training primarily focused on the methodology for each session, potential challenges they might encounter, and strategies to address them. At the end of the training, an assessment was conducted for all teachers to ensure they were adequately equipped to deliver the mental health intervention program. Moreover, during the intervention period, teachers formed teams that met once a week to discuss any challenges faced and collaboratively brainstorm solutions. Through these ways, we ensured that the teachers were well-prepared to deliver the intervention sessions, thereby enhancing the effectiveness of the intervention.

Stage 3: Post-intervention Assessment (June 2020). Following the intervention, all participants completed the same questionnaires once again. Due to illness or school transfer, 18 students were excluded from this study, resulting in a total of 1,001 valid responses. This included 475 from the intervention group and 526 from the control group.

### 2.4. Measures

#### 2.4.1. Life satisfaction

Children’s life satisfaction was assessed using an adapted Chinese version of the Multidimensional Student’s Life Satisfaction Scale (MSLSS; Huebner, 1994; Dong and Lin, 2011). This scale was designed to evaluate children’s satisfaction across various important domains in their lives, including school, family, friends, self, and environment. It consists of 25 items and is rated by a 4-point Likert scale ranging from 1 (strongly disagree) to 4 (strongly agree). The original MSLSS is well established (Huebner, 1994) and the Chinese version demonstrates satisfactory reliability and validity (Dong and Lin, 2011). In the present study, the Cronbach’s α coefficients for satisfaction with school, family, friends, self, and environment were 0.72, 0.74, 0.81, 0.80, and 0.68, respectively, at the baseline assessment, and were 0.76, 0.77, 0.83, 0.81, and 0.75, respectively, at post-intervention.

#### 2.4.2. Self-confidence

The Children’s Self-Confidence Questionnaire (Dong and Lin, 2011) was employed to measure the degree of self-confidence. This scale is composed of 17 items subdivided into three subscales: self-efficacy (6 items), self-assurance (7 items), and self-competence (4 items). A 4-point scale was used to rate each item, ranging from 1 (very inconsistent) to 4 (very consistent). Subscale scores were obtained by averaging item scores, with a higher score indicating a greater degree of self-confidence. This scale has shown satisfactory psychometric properties in previous studies (Dong and Lin, 2011). During the baseline assessment, the Cronbach’s α coefficients for self-efficacy, self-assurance, and self-competence were 0.77, 0.87, and 0.63, respectively. The coefficients were 0.80, 0.88, and 0.65, respectively, at post-intervention.

### 2.5. Data analysis

Preliminary analyses were conducted using SPSS 21 to assess the means, standard deviations, and potential group differences for the study variables. Subsequently, ANOVAs were employed to examine the mean differences in the study variables between baseline and post-intervention for both the intervention and control groups, aiming to evaluate the intervention effect.

## 3. Results

### 3.1. Characteristics of participants in the intervention and control groups

In the final sample, the intervention group included 250 boys and 225 girls (*M* = 11.57 years, *SD* = 1.082 years), and the control groups included 279 boys and 247 girls (*M* = 11.38 years, *SD* = 0.980 years). Table 1 presents additional characteristics of participants in the intervention and control groups.

**Table 1:** Basic information of the participants in the intervention and control groups.

	Intervention group (*n* = 475)	Control group (*n* = 526)	*p* value
Child sex, boys (girls)	250 (225)	279 (247)	0.97
Child age in years, mean (SD)	11.57 (1.34)	11.38 (1.53)	0.01
Grade, *n* (%)			
4	197 (41.47%)	229 (43.54%)	0.10
5	155 (32.63%)	181 (34.41%)	
6	123 (25.89%)	116 (22.05%)	
Area			
County JT	148 (31.16%)	98 (18.63%)	0.00
County JY	218 (45.89%)	291 (55.32%)	
County TL	109 (22.95%)	137 (26.05%)	
Maternal education, *n* (%)			
Primary school	106 (22.32%)	151 (28.71%)	0.70
Middle school	267 (56.21%)	235 (44.68%)	
High School	68 (14.32%)	87 (16.54%)	
College degree	34 (7.16%)	53 (10.08%)	
Paternal education, *n* (%)			
Primary school	85 (17.89%)	95 (18.06%)	0.97
Middle school	268 (56.42%)	278 (52.85%)	
High School	90 (18.95%)	112 (21.29%)	
College degree	32 (6.74%)	41 (7.79%)	
Sources of family income, *n* (%)			
Farming	160 (33.68%)	174 (33.10%)	0.78
Rural-to-urban migrant labor	277 (58.32%)	304 (57.79%)	
Others	38 (8.00%)	48 (9.12%)	

### 3.2. Differences in study variables between groups and time

Eight 2 (group: intervention vs. control groups) × 2 (time: pre- vs. post-intervention) ANOVAs with child age and gender as covariates were performed. Results showed that, among all 8 ANOVAs, 7 interaction effects were significant [for family satisfaction, *F*(1, 887) = 15.76, *p* < 0.001; for school satisfaction, *F*(1, 883) = 13.90, *p* < 0.001; for environmental satisfaction, *F*(1, 885) = 23.48, *p* < 0.001; for self-satisfaction, *F*(1, 886) = 32.36, *p* < 0.001; for self-efficacy, *F*(1, 887) = 33.39, *p* < 0.001; for self-assurance, *F*(1, 887) = 27.81, *p* < 0.001; for self-competence, *F*(1, 887) = 33.50, *p* < 0.001]. However, there was no significant interaction between group and time on friend satisfaction. Additionally, neither the main effect of the group nor the main effect of time was significant on all study variables (Table 2).

**Table 2:** Mean differences in study variables between baseline and post-intervention for the intervention and control groups.

Variables	Intervention group		Control group	
	Baseline *M* (*SD*)	Post-intervention *M* (*SD*)	Baseline *M* (*SD*)	Post-intervention *M* (*SD*)
Family satisfaction	3.56 (0.49)	3.70 (0.44)	3.53 (0.51)	3.51 (0.53)
School satisfaction	3.72 (0.42)	3.77 (0.40)	3.76 (0.36)	3.69 (0.41)
Environmental satisfaction	3.47 (0.47)	3.58 (0.46)	3.38 (0.51)	3.32 (0.57)
Self-satisfaction	3.23 (0.55)	3.44 (0.55)	3.27 (0.56)	3.23 (0.52)
Friend satisfaction	3.65 (0.46)	3.67 (0.46)	3.60 (0.47)	3.55 (0.54)
Self-efficacy	3.12 (0.57)	3.30 (0.53)	3.16 (0.55)	3.08 (0.56)
Self-assurance	3.13 (0.62)	3.34(0.62)	3.22 (0.63)	3.10 (0.65)
Self-competence	2.90 (0.65)	3.06 (0.59)	2.96 (0.64)	2.84 (0.64)

Results of further *post hoc* comparisons indicated that in the intervention group, the levels of family, school, environmental, and self-satisfaction showed a significant improvement post-intervention compared to baseline measurements. Moreover, there was a significant increase in levels of self-efficacy, self-assurance, and self-competence from baseline to post-intervention assessments.

Finally, *post hoc* comparisons showed that in the control group, there was a significant decrease in the levels of school, environmental, and friend satisfaction. Similarly, the levels of self-efficacy, self-assurance, and self-competence were significantly lower in post-intervention as compared to baseline assessments. However, the levels of family and self-satisfaction did not significantly change from baseline to post-intervention assessments.

## 4. Discussion

Utilizing a randomized controlled trial design, this study examined the effects of a mental health education intervention on life satisfaction and self-confidence among rural Chinese children. The findings revealed no significant differences in life satisfaction and self-confidence dimensions between the intervention and control groups during the baseline assessment. Following a 15-week intervention, a significant enhancement was observed in the levels of family, school, environmental, and self-satisfaction, as well as in self-efficacy, self-assurance, and self-competence among children in the intervention group. Conversely, for children in the control group, there was a decline in the levels of school, environmental, and friend satisfaction. Similarly, post-intervention measures of self-efficacy, self-assurance, and self-competence were significantly lower than their baseline levels for children in the control group.

The significant increase in the dimensions of life satisfaction and self-confidence post-intervention for the children in the intervention group lends support to the efficacy of such interventions, as evidenced in prior studies (Durlak et al., 2011; Weare and Nind, 2011; Merenda, 2021).

Children living in rural areas often grapple with a variety of stressors that significantly impact their mental health and overall well-being (Evans and Kim, 2013). According to Lazarus and Folkman (1984), stress emerges when an individual perceives a situation to exceed their resources. For children in rural areas, poverty is a prominent challenge, and the scarcity of resources—financial, educational, and social—exacerbates their stress, often leading to diminished life satisfaction and self-confidence (Yoshikawa et al., 2012; Maryani et al., 2018). Lazarus and Folkman (1984) further propose that individuals need to learn to reappraise their feelings positively to effectively manage stress. Such reappraisal can be facilitated through targeted interventions that equip children with the necessary skills to navigate their stressors.

Contrary to our hypothesis, friend satisfaction did not show a significant improvement by the end of the 16-week intervention. This may be due to the complex and somewhat resistant nature of peer relationships (Brown and Larson, 2009; Rubin et al., 2011). As children transition into adolescence, their relationships with peers become more complex and begin to play a more significant role in their lives (Marion et al., 2013). Influences such as the onset of puberty, an increase in personal autonomy, and shifts in social contexts complicate these relationships further (Schwarz et al., 2012). In our intervention, the four-week period allocated to improving peer relationships may not have been adequate to effect substantial change within this dynamic. Thus, future peer relationship interventions are encouraged to address the particular challenges to improve peer relationships (e.g., Durlak et al., 2011; Lund et al., 2018). Given the crucial role that peer relationships play in children’s early development (Marion et al., 2013; Parker et al., 2015), future interventions specifically designed to enhance peer relationships, such as fostering skills in conflict resolution, communication, and empathy, could offer more valuable insights.

Surprisingly, we found that compared to the baseline assessment, there were significant decreases in the levels of school, environmental, and friend satisfaction, as well as self-efficacy, self-assurance, and self-competence for children in the control group. These findings indicate a potential developmental decline in life satisfaction and self-confidence for children living in rural China. Limited access to resources and the pervasive stress associated with low SES may create a series of challenges that can obstruct psychosocial development, thereby eroding life satisfaction and self-confidence over time (Evans and Kim, 2013). Poverty can disrupt children’s daily routines, diminish their sense of security, and limit their access to positive experiences and opportunities (Yoshikawa et al., 2012). For children living in rural areas of China, these factors are often compounded by geographic isolation, poor quality of schooling, and reduced availability of community and social support services (Lund et al., 2018). Given the complex and multifaceted nature of these challenges, it is perhaps unsurprising that children from impoverished rural areas may experience a developmental decline in life satisfaction and self-confidence. This decline underscores the urgent need for interventions aimed at bolstering these areas of children’s lives. However, it’s worth noting that since we did not investigate the specific factors leading to the decrease in life satisfaction among children in the control group, we cannot establish a clear causal relationship. Future studies could delve deeper into this issue through longitudinal research.

In this study, we implemented a mental health education intervention aimed at teaching rural children how to effectively manage stressors associated with family dynamics, parental interactions, school environments, and peer relationships. By participating in this intervention, children from impoverished backgrounds residing in rural China may find it easier to reinterpret their life situations and assign them with a beneficial or positive meaning. Mental health education programs have the potential to mitigate some of these adverse effects. By equipping children with strategies to better understand and navigate their emotional experiences, these programs can assist children in reframing their life circumstances in a more positive light. It can promote adaptive coping mechanisms, resilience, and positive psychosocial development, even in the face of ongoing adversities (Durlak et al., 2011; Weare and Nind, 2011). Our findings serve as a stark reminder of the realities faced by many children in impoverished rural areas. Yet, they also underscore the potential for mental health education programs to make a significant difference. By instilling necessary coping skills, these interventions pave the way for children to lead more positive, meaningful lives, even amidst challenges. Most prior intervention studies have commonly selected a limited number of children through random sampling (Durlak et al., 2011). However, in this study, we implemented a school-based universal intervention across all primary schools governed by three rural counties, targeting all students from grades 4 to 6. Such an inclusive strategy has been proven to be effective in enhancing the overall school environment and boosting students’ quality of life.

### 4.1. Limitations and future directions

There are some limitations of the current study. First, due to challenges associated with longitudinal data collection, we only compared children’s life satisfaction and self-confidence at baseline and post-intervention. As such, the duration of the intervention’s effect remains uncertain. Future research should conduct more extended follow-ups to ascertain the long-term impact of such interventions. Second, our mental health education program took a relatively broad and general approach. Future studies could implement more targeted interventions, focusing on specific areas such as peer relationships or parent–child interactions. In addition, future research could leverage more specialized psychological intervention techniques. Techniques such as cognitive-behavioral therapy and mindfulness-based interventions, which have shown considerable promise in other contexts (Semple et al., 2010; Cheang et al., 2019), could potentially enhance the effectiveness of interventions for children living in rural and impoverished settings. Furthermore, since all of our data were reported by children and considering that children may not fully understand complex family economic situations, we did not collect information pertaining to specific family incomes and other factors indicative of the SES status of the families. Finally, future studies may benefit from conducting further interviews with students who participated in the intervention. This would help identify which aspects of the intervention that contributed to their positive changes. Such insights are valuable for refining and enhancing the interventions.

### 4.2. Implications

The findings from this intervention study underscore the importance of implementing targeted mental health education programs to improve life satisfaction and self-confidence among children in rural China. The success of this program in enhancing wellbeing highlights the need for policymakers to prioritize mental health initiatives in rural schools. Specifically, officials should fund the development and delivery of socioemotional learning programs tailored to impoverished areas (Jennings and Greenberg, 2009). Teachers require training, resources, and ongoing support to effectively educate students on managing emotions, building self-esteem, and fostering relationships (Wei and Kutcher, 2014). Schools may also implement policies and practices that create positive, supportive environments critical for mental health (Whitley et al., 2013). Counseling services and teacher-student mentoring programs are examples of initiatives that could complement curriculum-based interventions. Comprehensive efforts to improve mental health will empower rural Chinese youth to reach their full potential and interrupt cycles of poverty.

## 5. Conclusion

In this study, we utilized a randomized controlled trial design to assess the effectiveness of a mental health education intervention on life satisfaction and self-confidence among children in rural China. The findings demonstrated that after a 1-week intervention, significant improvements were observed in family, school, and environmental satisfaction, as well as in self-efficacy, self-assurance, and self-competence among children in the intervention group. In contrast, the control group exhibited declines in school, environmental, and friend satisfaction, along with reduced self-efficacy, self-assurance, and self-competence. This study highlights the crucial role of interventions to enhance positive development outcomes, such as life satisfaction and self-confidence, particularly among children residing in impoverished rural areas. We also provided some specific recommendations for policymakers and practitioners working with children in rural, impoverished areas.

## Data availability statement

The raw data supporting the conclusions of this article will be made available by the authors, without undue reservation.

## Ethics statement

The studies involving humans were approved by the Ethics Committee of the Department of Psychology, Beijing Normal University. The authors assert that all procedures contributing to this work comply with the ethical standards of the relevant national and institutional committees on human experimentation and with the Helsinki Declaration of 1975, as revised in 2008. The studies were conducted in accordance with the local legislation and institutional requirements. Written informed consent for participation was not required from the participants or the participants’ legal guardians/next of kin because during the implementation of the project, the teachers involved in the project fully communicated with the parents of students. The parents were fully informed.

## Author contributions

ZL: Writing – original draft, Conceptualization, Data curation, Formal analysis, Methodology. KW: Writing – original draft. HW: Writing – original draft. WY: Writing – original draft. CL: Data curation, Investigation, Project administration, Resources, Writing – review & editing.
